# LithoVue™ for renal stone therapy – a perfect fit for high volume academic centers; a retrospective evaluation of 108 cases

**DOI:** 10.1186/s12894-020-00624-3

**Published:** 2020-05-18

**Authors:** Maximilian Pallauf, Sabina Sevcenco, Christopher Steiner, Martin Drerup, Michael Mitterberger, Daniela Colleselli, Lukas Lusuardi, Thomas Kunit

**Affiliations:** 1 Landeskrankenhaus Salzburg - Universitätsklinikum der Paracelsus Medizinischen Privatuniversität, Universitätsklinik für Urologie und Andrologie, Müllner Hauptstraße 48, 5020 Salzburg, Austria; 2 Abteilung für Urologie und Andrologie, Sozialmedizinisches Zentrum Ost – Donauspital, Langobardenstraße 122, 1220 Wien, Austria

**Keywords:** Nephrolithiasis, Flexible ureterorenoscopy, Single-use flexible ureterorenoscope, LithoVue™

## Abstract

**Background:**

Over the last few years the number of flexible ureterorenoscopies, used for renal stone treatment, has risen steadily. This was associated with an increase in costs for maintenance and repair of the fragile ureterorenoscopes used. To overcome this problem single-use devices have been introduced to the market. The aim of this study was to assess surgical outcome and workability for LithoVue™, a single-use flexible ureterorenoscope.

**Methods:**

We retrospectively analyzed all flexible ureterorenoscopies performed at our department between January and October 2017. We included a total of 108 interventions for renal stone therapy, all performed using the single-use device LithoVue™. We assessed patients’ characteristics including stone size, count and location. We evaluated the surgical outcome, analyzing stone-free rates, reintervention rates, complication rates, as well as surgery time. Learning curve for single-use ureterorenoscopes was evaluated by comparing the surgical outcome between residents and consultants.

**Results:**

The average time needed per intervention was 52,31 min ± 28,11. In 77 out of 108 (71,30%) patients we were able to remove all stones by a single intervention. In 8 patients (7,41%) intra- or postoperative complications occurred, none of which was graded higher than Clavien-Dindo III B. We did not find any statistical differences comparing the surgical outcome between residents and consultants. No technical difficulties occurred during surgery.

**Conclusion:**

Single-use flexible ureterorenoscopes provide decent working properties resulting in good surgical outcome. Furthermore, they are proven to be easy to handle even for unexperienced surgeons, making them a feasible choice for high volume academic centers.

## Background

Over the last decade flexible ureterorenoscopy became more and more popular for upper urinary tract stone treatment, leading to numerous innovations in the field of flexible ureterorenoscopy. Several different types of flexible ureterorenoscopes are available, using fiber optics or digital imaging for image transmission. Whereas digital ureterorenoscopes have shown to have better image quality [1], fiber optic ones have better end-tip deflection providing better access to sharp angled calices [2]. As numbers of flexible ureterorenoscopies are constantly rising [3], costs for flexible ureterorenoscopy have become of great importance for health care providers. High costs per surgery can be explained by the purchase price of the flexible ureterorenoscope as well as high maintenance costs, including sterilization and repair. Since durability of reusable flexible ureterorenoscopes is low, needing substantial repair every 6 [4]-31 [5] interventions, single-use flexible ureterorenoscopes have been introduced.

There are several different single-use flexible ureterorenoscopes available. The most tested one is LithoVue™, a digital flexible ureterorenoscope manufactured by Boston Scientific®, showing similar working properties as well tested reusable ones [6–8]. Whereas maneuverability as well as image quality of LithoVue™ have been proven not to be inferior to those of reusable ones, [9] it is still debatable whether single-use flexible ureterorenoscopes are more cost-effective than their reusable counterparts. Several previous studies compared the costs for both instruments, showing that single-use flexible ureterorenoscopes are only cost-effective for institutions where less than 99–118 interventions are being performed per year [10, 11]. Nevertheless, the authors do not take into consideration that repair rates for reusable ureterorenoscopes might differ depending on the type of institution where they are being used. For example, it seems plausible that hospitals that fulfill a training assignment and teach young doctors might experience a higher rate of instrument breakage than centers where only experienced surgeons perform flexible ureterorenoscopy.

In our department we experienced a high number of instrument breakage using reusable ureterorenoscopes. Whether this was due to inappropriate handling intraoperatively or postoperatively was not clear. However, considering that approximately one third of all flexible ureterorenoscopies had been carried out by a resident, introduction of a single-use device appeared to be a reasonable choice. Following one year of experience in using LithoVue™ we finally wanted to evaluate its impact on renal stone treatment and verify whether it had any effects on residents’ surgical skills. Therefore we retrospectively assessed surgical outcome and complications in all flexible ureterorenoscopies performed between January and October 2017.

## Methods

We retrospectively reviewed all flexible ureterorenoscopies performed at our department between January and October 2017. Data was collected from patient medical records. Personal data was encrypted. Since this is a retrospective study, no patient consent could be obtained in advance. Permission to collect the data was given by the department. We only included flexible ureterorenoscopies performed for renal and ureteral stone therapy using the single-use device LithoVue™. We excluded all diagnostic flexible ureterorenoscopies or flexible ureterorenoscopies performed for upper tract urothelial carcinoma (UTUC) therapy. Age, gender, comorbidities, health status or long-term medication were not part of the exclusion criteria. Since we do not treat children in our department, all patients were of age. A total of 108 flexible ureterorenoscopies were found to be eligible for our study.

We examined whether the surgery was performed by a resident or a consultant and recorded surgery time of every intervention. Furthermore, we assessed stone size, count and location in all patients using preoperative computerized tomography (CT) scans and surgery reports. Patients were considered to be free of renal stones when no residuum was found either by endoscopic inspection of the pelvicalyceal system or in the retrograde pyelogram at the end of the intervention. In addition, we recorded whether ureteral stones were present and whether they had been removed. Furthermore, we recorded whether a laser was needed for fragmentation of large stones. Also, we noted if any intraoperative complication occurred. The information mentioned above, except for preoperative stone size, location and count, was taken from the surgery report. By additionally studying the patients’ hospital record, we revealed whether a patient needed any further surgeries for renal stone therapy or if any postoperative complication occurred. The decision whether a patient needed a second intervention was made on a case to case basis. As this is a retrospective study there was no form where physicians declared their reason for planning reintervention. Therefore, we cannot provide more precise information on this. If a complication occurred, regardless of whether this was noticed intra- or postoperatively, it was classified according to the Clavien-Dindo (CD) classification.

Furthermore, we investigated whether any statistically significant differences can be found comparing surgery time, complication rates, stone-free rates and rates of reintervention between residents and consultants. To prevent any statistical bias caused by differences in stone size and count we compared stone characteristics between residents and consultants as well.

For comparing surgery times and differences in stone size and count we used Mann-Whitney-U Test or students T-Test depending on whether the samples were normally distributed. Study samples were tested for normal distribution using Shapiro-Wilks test. To evaluate the effect size of the tests, we calculated Cohen’s d. For comparing complication rates, stone-free rates and rates of reintervention we used Fisher’s exact test. Odd’s ratio was calculated to evaluate the tests’ effect size.

Differences were considered to be statistically significant at *p* < 0.05. All statistical analyzes were performed using XLSTAT by Addinsoft.

## Results

### Patient characteristics, surgery specific characteristics and stone characteristics

Patient characteristics, surgery specific characteristics and stone characteristics are listed in Table 1.

**Table 1:**
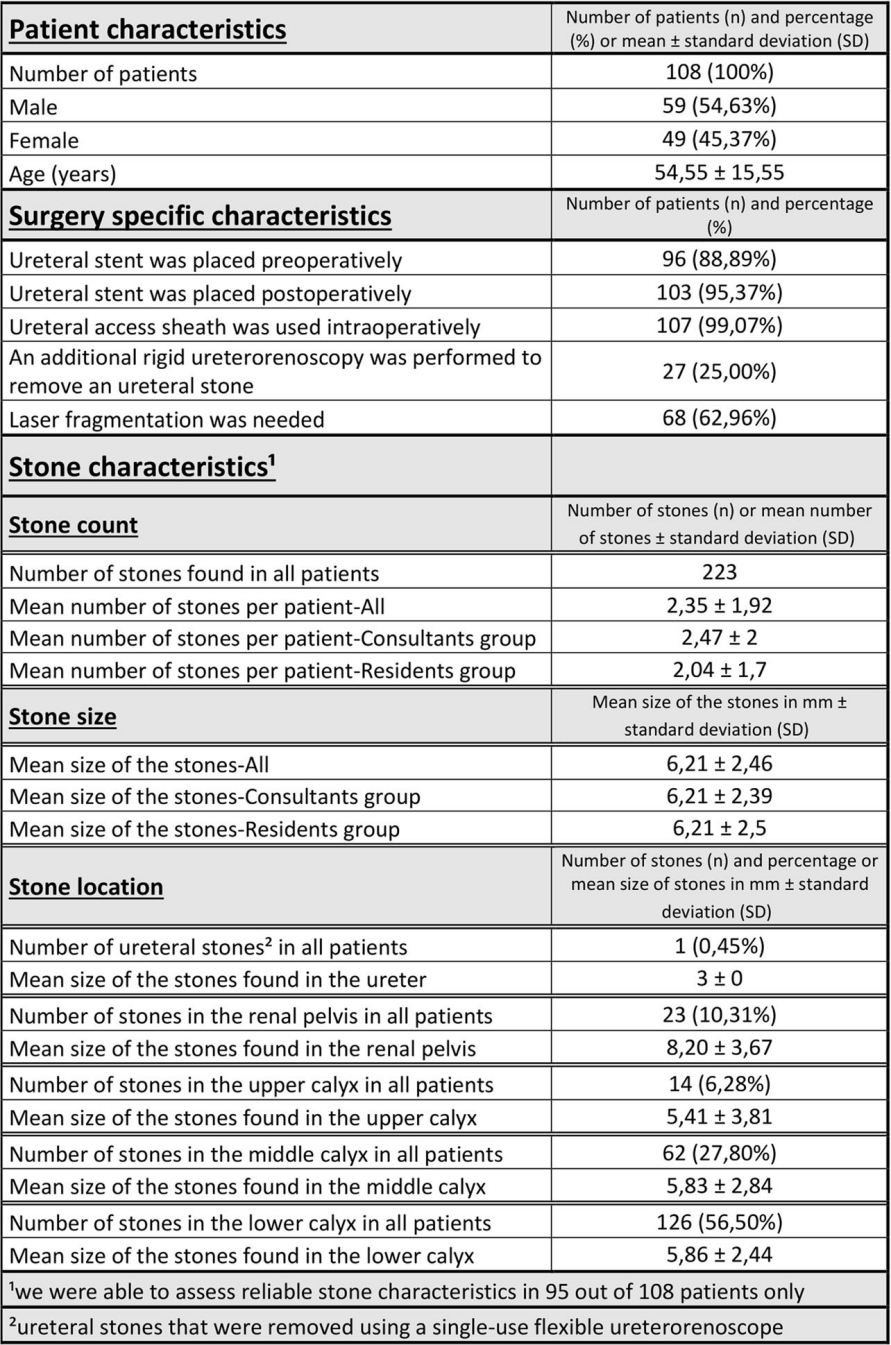
Patient characteristics, surgery specific characteristics and stone characteristics. The first part of the table gives information about general characteristics of the patients such as gender and age. The information on gender distribution is given in absolute numbers and percentages in relation to the total number of patients. The average age of the patients is given in years and is represented by the mean value together with the standard deviation. The second part of the table summarizes surgery specific characteristics for example in how many patients an ureteral stent was placed pre- and postoperatively, whether an access sheath was needed, whether an additional rigid ureterorenoscopy was performed in the same surgery and whether a laser was needed for stone fragmentation. The information is given in absolute numbers and percentages in relation to the total number of patients. The third part of the table provides detailed information on the characteristics of the patients’ renal stones. The total number of renal stones found in all patients is given as well as the average number of stones found per patient and group. The latter is represented by the mean value together with the standard deviation. Furthermore, it provides an overview on the local distribution of the stones in the pelvicalyceal system (ureter, renal pelvis, upper calyx, middle calyx, lower calyx). For each location the number of stones found as well as their percentage in relation to the total number of stones are given. Additionally, the table provides information on the average size of the renal stones found and is given for each group and each location in the pelvicalyceal system. Average stone size is given in millimeters and is represented by its mean value together with the standard deviation.

### Results of surgery

Results of surgery are listed in Table 2.

**Table 2:**
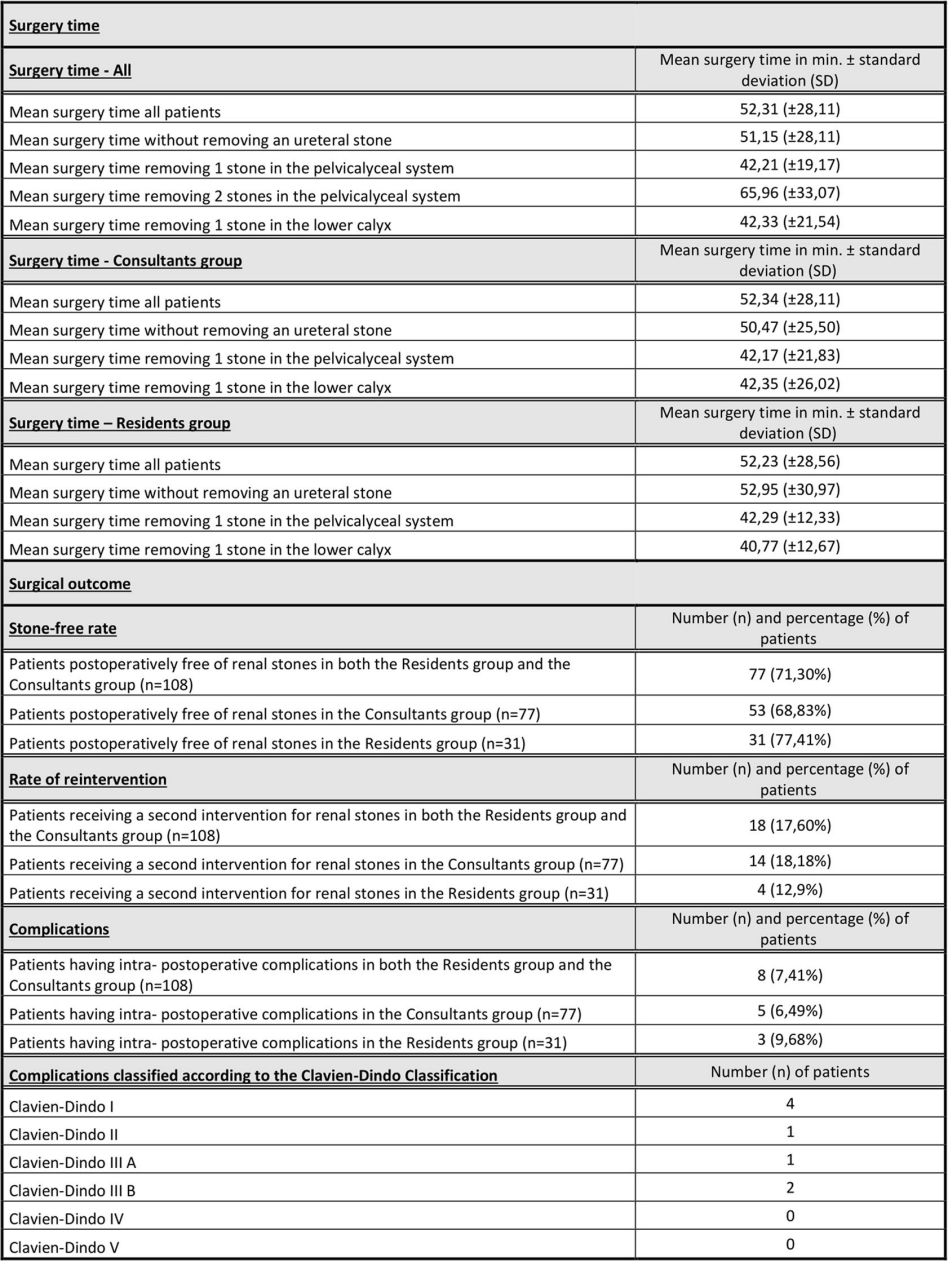
Results of surgery. The first part of this table provides detailed information on the time needed to perform a flexible ureterorenoscopy. The mean surgery time for each group (all, consultants and residents) is given in minutes together with the standard deviation. The second part of this table summarizes the surgical outcome. For each event (stone-free rate, rate of reintervention and complications) and each group (all, consultants and residents) the number of patients as well as their percentage in relation to the total number of patients are given. Furthermore, complications are classified according to the Clavien-Dindo Classification. For each grade the total number of patients is given.

### Complications

In 8 out of 108 patients (7,41%) an intra- or postoperative complication occurred, none of which was graded higher than CD III B. Four times leakage of contrast media from the efferent urinary tract into the retroperitoneal space was observed intraoperatively, leading to a prolonged course of urethral stenting (CD I; Residents 2, Consultants 2). One patient suffered of pyelonephritis due to a subcapsular hematoma. The patient had to be treated with antibiotics intravenously (CD II; Residents 1, Consultants 0). No surgical intervention was necessary. In one patient (female) the ureteral stent was dislocated and needed to be relocated under local anesthesia (CD III A; Residents 0, Consultants 1). Two patients needed an intervention under general anesthesia due to postoperative complications (CD III B; Residents 0, Consultants 2). One patient (male) developed high fever after the ureteral stent was removed. A new ureteral stent had to be inserted, therefore needing general anesthesia. One patient developed ureteral stenosis following flexible ureterorenoscopy needing temporary ureteral stenting and laparoscopic surgery of the ureter.

In 68 out of 108 patients (62,96%) a laser was needed to fragment renal stones. The use of a laser did not increase the risk for intraoperative complications (7,35% vs.7,5%; *p* = 1).

### Resident versus consultant

#### Surgery time

**Fig. 1:**
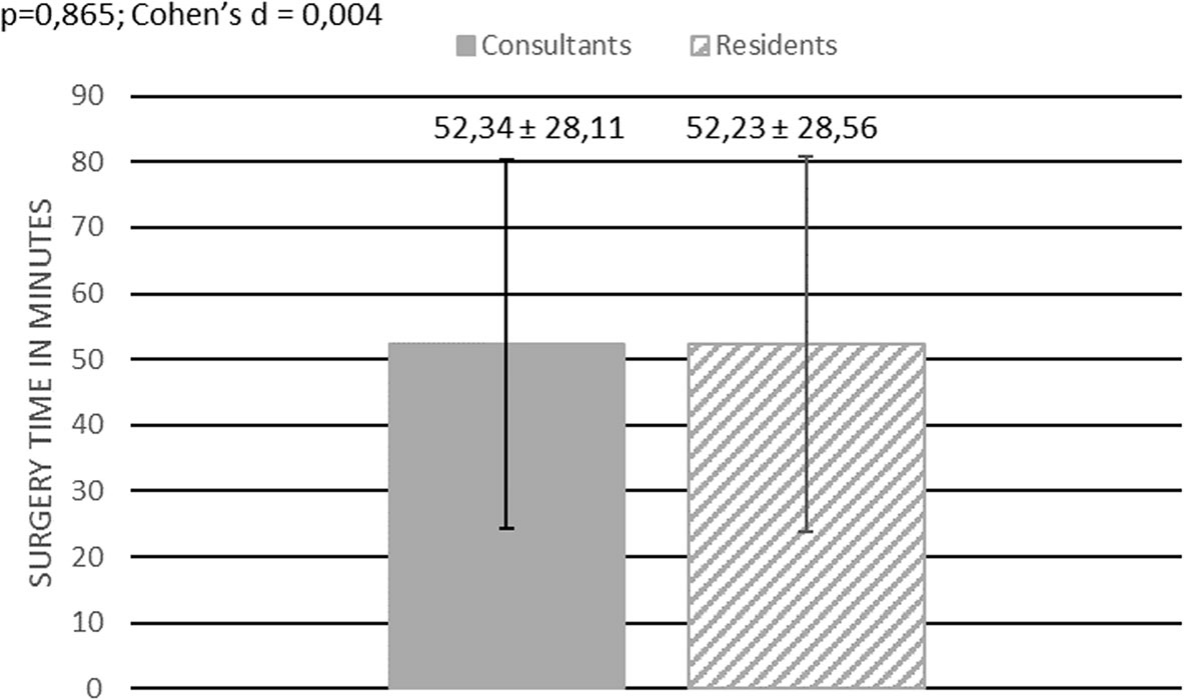
Surgery time – all patients. This figure shows a bar chart comparing surgery time between consultants and residents. Surgery time is given in minutes. The dark grey box indicates mean surgery time for consultants, the grey and white stripped box indicates mean surgery time for residents. The vertical black line indicates the standard deviation for each group. Additionally, the mean surgery time including the standard deviation is shown for both groups. The result of the statistical comparison between both groups is shown in the left upper corner (*p*-value, Cohen’s d)

**Fig. 2:**
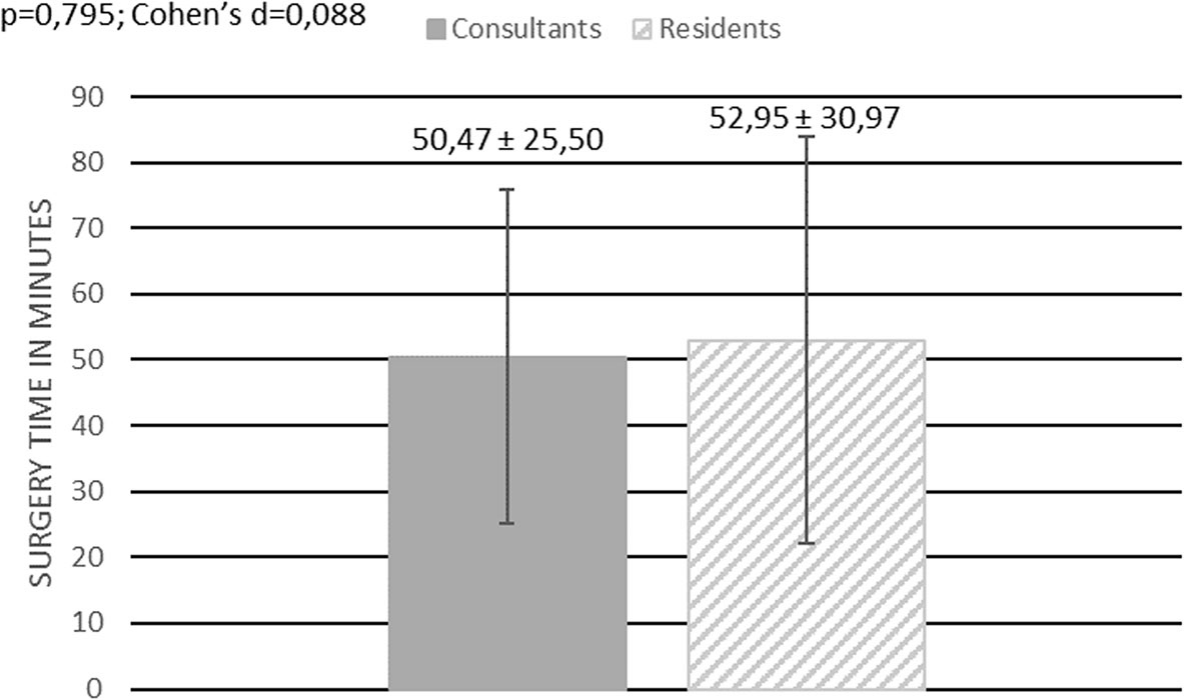
Surgery time – all patients without an ureteral stone. This figure shows a bar chart comparing surgery time between consultants and residents in patients where solely stones in the pelvicalyceal system have been removed. Surgery time is given in minutes. The dark grey box indicates mean surgery time for consultants, the grey and white stripped box indicates mean surgery time for residents. The vertical black line indicates the standard deviation for each group. Additionally, the mean surgery time including the standard deviation is shown for both groups. The result of the statistical comparison between both groups is shown in the left upper corner (*p*-value, Cohen’s d)

**Fig. 3:**
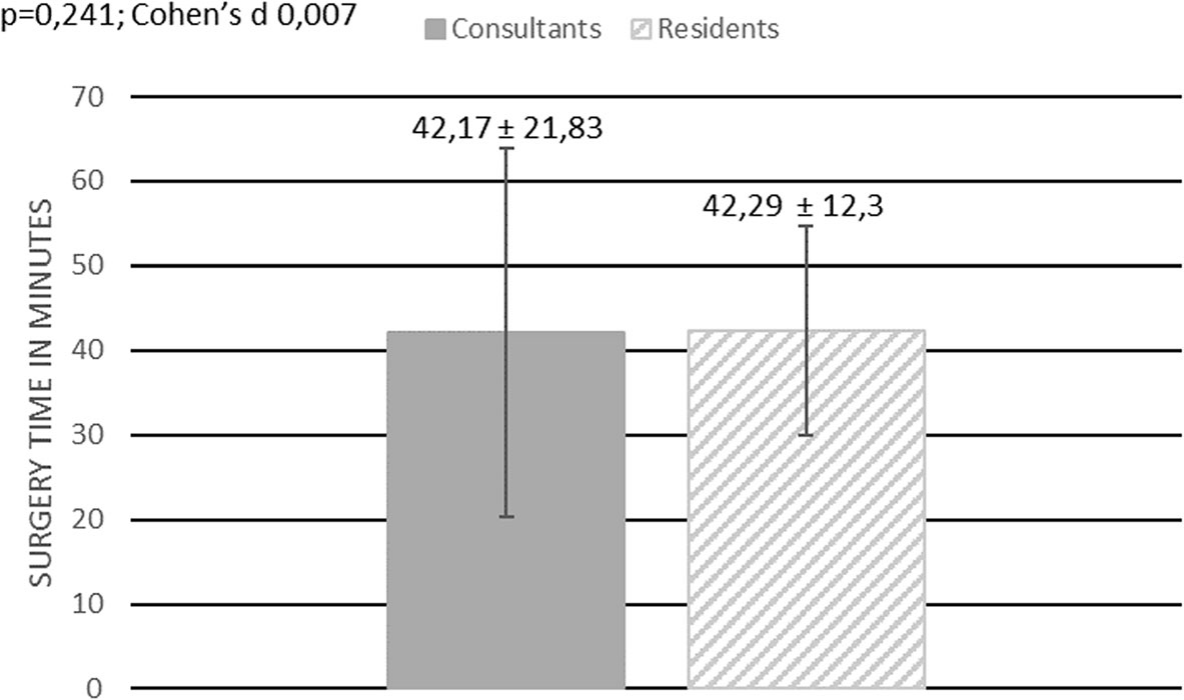
Surgery time – all patients with only one kidney stone removed. This figure shows a bar chart comparing surgery time between consultants and residents in patients where only one kidney stone has been removed. Surgery time is given in minutes. The dark grey box indicates mean surgery time for consultants, the grey and white stripped box indicates mean surgery time for residents. The vertical black line indicates the standard deviation for each group. Additionally, the mean surgery time including the standard deviation is shown for both groups. The result of the statistical comparison between both groups is shown in the left upper corner (*p*-value, Cohen’s d)

**Fig. 4:**
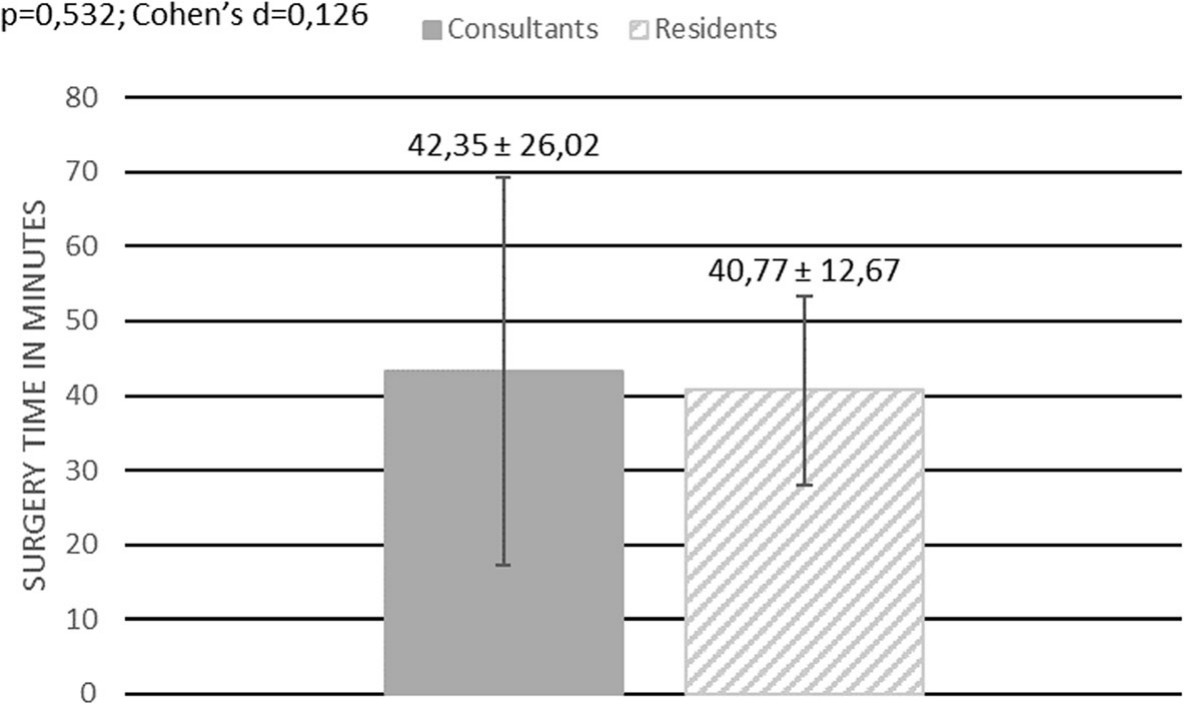
Surgery time – all patients with only one stone in the lower calyx. This figure shows a bar chart comparing surgery time between consultants and residents in patients when solely one stone in the lower calyx has been removed. Surgery time is given in minutes. The dark grey box indicates mean surgery time for consultants, the grey and white stripped box indicates mean surgery time for residents. The vertical black line indicates the standard deviation for each group. Additionally, the mean surgery time including the standard deviation is shown for both groups. The result of the statistical comparison between both groups is shown in the left upper corner (*p*-value, Cohen’s d)

108 interventions have been included in our study. 77 interventions were performed by a consultant, 31 by a resident. The average time needed to perform a flexible ureterorenoscopy was 52,31 min ± 28,11. The average surgery time for a consultant was 52,34 min ± 28,11 and 52,23 min ± 28,56 for a resident, showing no statistically significant difference (*p* = 0,865; Cohen’s d = 0,004) Fig. 1**.** In 27 out of 108 surgeries an additional rigid ureterorenoscopy was performed in order to remove an ureteral calculus. The average surgery time was 55,78 min ± 31,27. The average surgery time for patients without an ureteral calculus was 51,15 min ± 26,85. No statistically significant difference in surgery time was found for those patients comparing surgery time between residents, 52,95 min ± 30,97, and consultants, 50,47 min ± 25,50 (*p* = 0,795; Cohen’s d = 0,088) Fig. 2. Furthermore, we investigated whether it took residents longer than consultants to perform a flexible ureterorenoscopy depending on the location of the renal calculus. No difference in surgery time between residents and consultants was found when stones were located only at one site of the pelvicalyceal system (renal pelvis, upper calyx, middle calyx, lower calyx) (*p* = 0,241; Cohen’s d 0,007) Fig. 3. The average time needed to remove stones by flexible ureterorenoscopy solely at a single site of the pelvicalyceal system was calculated to be 42,29 min ± 12,3 for residents, 42,17 min ± 21,83 for consultants, and 42,21 min ± 18,99 for consultants and residents together. Hence 56,5% of all removed stones were located in the lower calyx, we calculated the time needed to remove solely stones in the lower calyx. The average time needed was 42,33 min ± 21,54. It took residents 40,77 min ± 12,67 and consultants 42,35 min ± 26,02 to remove solely one stone from the lower calyx, showing no statistically significant difference (*p* = 0,532; Cohen’s d = 0,126) Fig. 4.

#### Stone-free rate, rate of reintervention, complications

To assess surgical outcome, we measured stone-free rates, rates of reintervention and complication rates. Following flexible ureterorenoscopy 77 out of 108 patients (71,3%) were considered radiologically or endoscopically free of renal stones. When comparing stone-free rates between residents (77,41%) and consultants (68,83%) we did not find any statistically significant difference (*p* = 0,482). We found an odds ratio of 1,55, favoring stone-free rates for residents.

18 out of 108 patients (17,6%) needed at least one more surgical intervention to be considered free of renal stones. As for stone-free rates, no statistical difference was discovered when comparing rates of reintervention between residents (12,9%) and consultants (18,18%) (*p* = 0,582). However, we found an odds ratio of 0,67 favoring a lower likelihood of reintervention for residents.

More severe complications (>CD II) were found in the consultants’ group. Hence this group made up for 71,3% of the cases this does not surprise. However, when comparing complication rates between residents (9,68%) and consultants (6,49%), we did not find a statistically significant difference (*p* = 0,687). We found an odds ratio of 0,65 favoring a lower risk for complications for consultants. An overview of the results is given in Table 3.

**Table 3:**
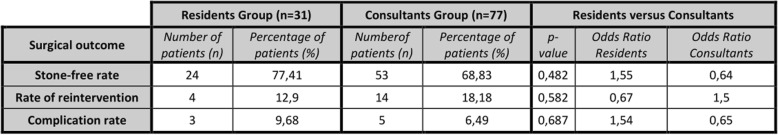
Surgical outcome – comparison between consultants and residents. This table provides detailed information on the statistical evaluation of the surgical outcome between consultants and residents. Surgical outcome was evaluated by comparing the stone-free rate, the rate of reintervention and the rate of complications between residents and consultants. For each event (stone-free rate, rate of reintervention, rate of complication) and each group (consultants and residents) the number of patients (n) meeting the criteria as well as their percentage in relation to the total number of patients for each group, are given. Differences between both groups (residents and consultants) on each event (stone-free rate, rate of reintervention, rate of complication) were calculated using Fisher’s exact test. The odd’s ratio was calculated to evaluate the tests’ effect size. For each event the *p*-value and the odds ratio for both groups (residents and consultants) are given.

#### Technical difficulties

No technical difficulties occurred.

## Discussion

In recent years flexible ureterorenoscopy has constantly gained in importance for renal stone therapy [3]. One probable reason therefor are the many improvements made for flexible ureterorenoscopes. As a matter of fact, rising numbers of flexible ureterorenoscopies promote the need for cost-effective devices. However, reusable digital or fiber optic flexible ureterorenoscopes demand high maintenance costs. This is partly due to the costs for sterilization but mainly due to the need for regular repairs. Several studies analyzed the average amount of interventions which can be performed before substantial repair is needed. Depending on the ureterorenoscope in use, repairs were necessary every 6 [4] to 31 [5] interventions. However, the large variance of the study results can not only be attributed to differences in the manufacturing quality of the devices. This can also be partly explained by the inaccuracy of the selected measurand. Because it is not the number of interventions, but rather the absolute operating time that influences the ureterorenoscopes’ durability. As the latter accumulates, the risk of equipment damage increases as well [12]. Still, there are also several other risk factors promoting ureterorenoscope damage. The most important ones are found to be a steep infundibulopelvic angle (IPA) [13] and the use of an ureteral access sheath (UAS) [14]. Steep IPA varies among patients and is often difficult to assess preoperatively. The use of an UAS increases irrigation flow while additionally reduces intrapelvic pressure and therefore is used routinely [15]. This illustrates that scope breakage is difficult to prevent, as risk factors can hardly be avoided in clinical practice. This applies above all to tertiary and reference centers as well as clinics that fulfil a training assignment. Especially for the latter, as prolonged surgery time, that might be due to teaching purposes, bears the risk of promoting ureterorenoscope damage [12]. To make matters worse, refurbishment of the devices seems to further increase their vulnerability and intensify this problem. Carey et al. showed, that once an reusable flexible ureterorenoscope undergoes substantial repair, an additional repair was needed much more often [16]. This again underlines the need for a robust and cost-effective alternative to reusable flexible ureterorenoscopes.

To address this problem, single-use flexible ureterorenoscopes have been introduced in 2009 [17]. Since the time they first became available, investigators analyzed their working properties in vitro [1, 7, 18] and in vivo [9], attesting single-use flexible ureterorenoscopes similar maneuverability and image quality as reusable ones. Furthermore, a recently published systematic review demonstrated that surgical outcome and complication rates do not differ between single-use and reusable flexible ureterorenoscopes [19].

Our study confirms the high functionality of disposable devices in stone therapy as its results are comparable to those found in the literature. In a large meta-analysis Davis et al. showed that rate of stone clearance and rate of complication for single-use flexible ureterorenoscopes are 87% ± 15 and 9,3% ± 9 respectively [19]. In all those studies, included in the meta-analysis, an average of 66 min ± 29 was needed to remove a single stone measuring 1,13 cm ± 0,26 cm [19]. In our study, stone clearance was reached in 71,3%, leading to only 17,6% of patients needing an additional treatment. Complications only occurred in 7,41% of interventions, none of which was graded higher than CD III B. A mean surgery time of 52,31 min ± 28,11 was needed to treat in average 2,35 ± 1,92 stones per patient, each calculated to be 6,21 mm ± 2,46 in diameter. When all patients were excluded who additionally received a rigid ureterorenoscopy, the mean surgery time was calculated to be as low as 51,15 min ± 26,85. Therefore we conclude, that introduction of single-use flexible ureterorenoscopes did not deteriorate surgical outcome at our department.

Since 28,7% of all interventions were performed by a resident, we tried to figure out whether the use of single-use flexible ureterorenoscopes worsens surgical outcome or prolongs surgery when used by an unexperienced surgeon. Therefore, we compared complication rates, stone-free rates and rates of reintervention between residents and consultants, showing no statistically significant difference. Furthermore, no statistical difference was found comparing surgery time between residents and consultants. This leads to the assumption, that single-use flexible ureterorenoscopes are easy to handle, also for less experienced surgeons. This is highlighted by the fact, that stone size and stone count did not differ between residents and consultants. Easy maneuverability might be partly due to the low weight of disposable ureterorenoscopes. As they are not meant to last for several interventions, less robust, but in fact, much lighter products can be used, thereby reducing physical effort [20].

However, the informative value of this study is limited due to its retrospective approach. In order to compare the user-friendliness between single-use and reusable flexible ureterorenoscopes a prospective randomized trial will be needed.

Even more controversial than the debate about the functionality of disposable devices is the debate about their cost efficiency. The question remains whether disposable devices are just an overpriced and unnecessary tool, or whether they can really help to reduce the increasing costs in stone therapy. Therefore, several investigators analyzed the costs for reusable ureterorenoscopes (purchase, sterilization, repair) and compared them with those (purchase) for single-use ones. As expected, their projected costs per intervention for reusable scopes differed significantly, ranging between 436$ (393,13€ - 01/2020) [11] and 848,1$ (769,22€ - 01/2020) [10], excluding costs of acquisition. This gap can be explained by the different number of repairs as well as costs for maintenance, storage and sterilization of the devices. This highlights the multiple difficulties, when trying to compare costs per intervention. However, the frequency and the extent of the repairs account for the largest amount and can therefore be used as a benchmark for cost comparison. In other words, if the repair rates are the same than this will be true for the operating costs as well.

As for our department, we experienced high rates of ureterorenoscope breakage prior to the introduction of single-use devices. In 2016 when we were still using solely reusable flexible ureterorenoscopes, substantial repair was needed every 9,2 interventions. This is similar to the rates that have been used in the previously mentioned studies to calculate operating costs for reusable devices 7,5 [11] – 12,5 [10]. By taking over their operating costs we can calculate the costs that would have incurred if we had performed the 108 interventions using a reusable instead of a disposable ureterorenoscope. The calculation is as follows: Due to the high number of cases we are covering every year, six reusable flexible ureterorenoscopes are in use in our department. The acquisition costs per device were 18544,99$ (16720€ - 01/2020) amounting to a total of 111269,93$ (100320€ - 01/2020). If the lower of the two reference values $436 (393,13€ - 01/2020) [11] is used to calculate the devices operating costs, this results in additional expenses of 47088$ (42457,98€ - 01/2020) for 108 interventions. Thus, the theoretical costs for 108 interventions using a reusable flexible ureterorenoscope sum up to 158357,93$ (142775,51€ - 01/2020) which is 1466,28$ (13220€ - 01/2020) per procedure. This amount roughly corresponds to the usual sales price for LithoVue™, which is 1500$ (1360,49€ - 01/2020) [21]. Therefore we can conclude that the use of LithoVue™ is also a cost-effective choice for our department. However, the final proof is still pending.

## Conclusion

We were able to show that the introduction of single-use flexible ureterorenoscopes at our department neither did deteriorate surgical outcome for renal stone therapy nor did it raise costs per intervention. Furthermore, single-use devices are proven to be easy to handle even for unexperienced surgeons, making them a feasible choice for high volume academic centers.

## Data Availability

The datasets used and/or analysed during the current study are available from the corresponding author on reasonable request.

## Abbreviations

CD: Clavien-Dindo
CT: Computerized tomography
IPA: Infundibulopelvic Angle
n: Number
SD: Standard Deviation
UAS: Ureteral Access Sheath
UTUC: Upper Tract Urothelial Carcinoma
